# 

**Published:** 1888-08

**Authors:** 


					﻿AXJO-XJST, 1888.
OLD SERIES
YokXXV
1858?^”'
NEW SERIEI
Vet. V.-K..S.

^’^W.F.WESTMORELAND.M d91
H.V.M.MILLER.M.D1.L.D^^M
^uBIJSMED By «nME8PJ4ARRISQN&CQ«
______________aELTLANTA,CA. SH
SUBSOttimONi MJK> A YEAR.
				

